# Gain of Imaging Fidelity by Employing a Higher Number of Independent Transmit Channels Together with Slice-Selective Radio-Frequency (RF) Shimming at 7T

**DOI:** 10.3390/ma7010030

**Published:** 2013-12-20

**Authors:** Niravkumar Darji, Gopesh Patel, Oliver Speck

**Affiliations:** 1 Department of Biomedical Magnetic Resonance, Otto-von-Guericke University Magdeburg, Leipziger Straße 44, Magdeburg 39120, Germany; E-Mails: gopesh.patel@ovgu.de (G.P.); oliver.speck@ovgu.de (O.S.); 2 German Centre for Neurodegenerative Diseases (DZNE), site Magdeburg, Magdeburg 39120, Germany; 3 Leibniz Institute for Neurobiology, Magdeburg 39118, Germany

**Keywords:** image homogeneity, radio frequency shimming, RF shimming, RF-power control, transmit array, multi-slice, 7T

## Abstract

Dielectric resonance effects and radio-frequency (RF) power deposition have become challenging issues for magnetic resonance imaging at ultrahigh-field (UHF) strengths. The use of transmit (Tx) coil arrays with independently-driven RF sources using a parallel transmission system is a promising method for alleviating the resulting RF inhomogeneities. In this study, the effect on homogeneity and RF-power when employing a higher number of transmit channels with multi-slice acquisition *in vivo* at high field strength (7T) is scrutinized. An 8-channel head coil array was driven to emulate circular polarized (CP) and 2-, 4-, and 8-channel independent transmit configurations at 7T. Static RF shimming was employed on human subjects in order to homogenize the B_1_^+^ field in the excited volume. Slice-selective and global RF shimming methods were applied with CP and 2-, 4-, and 8-channel transmit channel configurations. RF shimming was performed from CP to 2-, 4-, and 8-channel Tx configurations globally and slice-selectively. Systematic improvement in B_1_^+^ homogeneity and/or reduction in RF-power were observed. RF shimming in the human brain with 8-channel transmit and slice-selective shimming yields an increase in B_1_^+^ homogeneity of 43% and/or reduces RF-power by 68% when compared with CP global RF shimming at 7T.

## 1. Introduction

Magnetic resonance imaging of human subjects at high field strengths (≥3 T) suffers from radio frequency (RF) field inhomogeneity artifacts caused by dielectric resonance effects. The circularly polarized (CP) transmit B1 (B_1_^+^) field that creates the tipping of the magnetization produces variable tissue contrast depending on the geometry and the magnetic field strengths, while the inhomogeneity of the signal reception field (B_1_^−^) gives rise to variable signal reception sensitivity. While this latter effect is correctable given the coil sensitivities, the former effect is not readily correctable [1,2].

Presently, various methods are proposed for reducing transmit B1 field (B_1_^+^) inhomogeneity; many of them are already implemented on current imaging systems, e.g., the use of composite pulses [3] and adiabatic pulses [4]. Another alternative is the use of multiple transmit channels, which can be implemented in combination with the previously mentioned methods [5]. This technique requires a substantial additional investment in hardware. The basic idea behind the multi-channel transmit concept is to geometrically break the transmit field into multiple regions, where each region is controlled independently, thus creating spatial degrees of freedom that allow the delivery of specific excitation pulses from individual transmit channels. The parallel transmit hardware allows channel specific control over RF amplitudes and excitation phases that can mitigate high field B_1_^+^ inhomogeneity either globally or for isolated target anatomy [6]. This concept is known as B_1_^+^ shimming, or static RF shimming [2,7–9].

Systematic studies on RF shimming have primarily examined two independent transmit channels [10], but nowadays RF shimming on eight or more independent transmit channels has been studied [11–13]. In an effort to increase the number of degrees of freedom available for such RF field shaping, there has been a trend towards the use of a larger number of transmit channels, thus mirroring the development of parallel reception architecture in the past decade [9,11,14–16].

In this contribution, we investigated the gain in brain imaging fidelity (improvement in B_1_^+^ homogeneity and reduction in RF-power) by performing multi-slice MRI acquisitions with a standard pseudo-CP mode, and with higher numbers of independent transmit (Tx) channels (2, 4, and 8) [17,18], using slice-selective and global RF shimming [19] at 7T. The benefit of segmenting the volume into several slices was to reduce the RF shim region of interest (ROI) from the whole brain to each individual slice, thereby producing a series of simpler optimization problems to yield shim solutions with higher homogeneity [19]. The goal of this study was to experimentally demonstrate the possibility of trading homogeneity for RF-power and/or vice versa, for different numbers of Tx channels and shimming methods.

## 2. Results and Discussion

**Figure 1.**
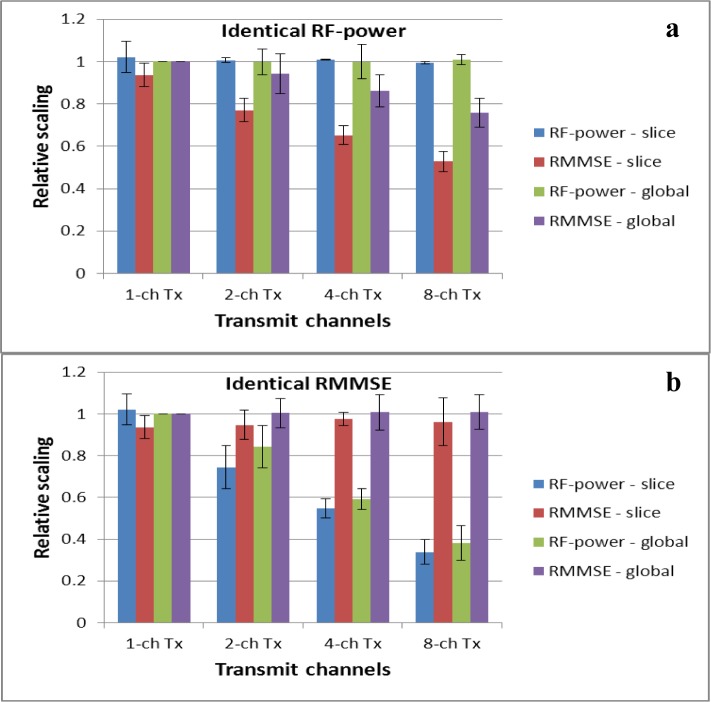
Radio-frequency (RF)-power and root magnitude mean square error (RMMSE), averaged over the volume, for the two shimming methods: slice-selective RF shimming and global RF shimming, for 1, 2, 4, and 8 Tx channels. (**a**) Represents findings when the RF-power was identical for all configurations and (**b**) shows findings when the RMMSE was identical for all configurations. All values have been normalized to the 1-channel Tx mode of the global RF shimming. Error bars represent the standard error over the subjects.

All experiments were performed on four healthy volunteers and had the aim of improved brain imaging. In this study, the CP mode is referred to as 1-channel Tx. In the case of 1-channel Tx, an identical RF-amplitude was applied for the whole slice stack in global RF shimming or for individual slices in slice-selective RF shimming, with CP phases, scaled to reach the desired mean flip angle (FA) over the target region. Figure 1 represents a simulation study of the gain in homogeneity at identical average RF-power (Figure 1a) and the reduction in RF-power at identical average homogeneity (Figure 1b) for the 1-, 2-, 4- and 8-channel Tx configurations over all slices and subjects. It includes findings from slice-selective and global RF shimming (normalized to the 1-channel Tx mode of global RF shimming) which depict the average values for different subjects, and the error bar represents the standard error over subjects. Both figures quantify the homogeneity by means of root magnitude mean square error (RMMSE) of the calculated B_1_^+^ field, and the RF-power is computed as the sum of squares of RF pulse amplitudes for all transmit channels. When slice-selective RF shimming was used instead of global RF shimming, and with an increasing number of transmit channels from 1-channel Tx to 8-channel Tx, the B_1_^+^ field distribution in the brain at 7T became more homogenous, and when all shim solutions had approximately identical homogeneity (Figure 1b), the RF-power progressively decreased with 8 numbers of Tx channels.

**Figure 2.**
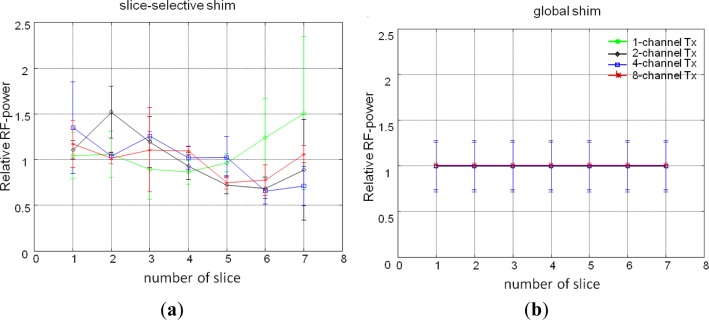

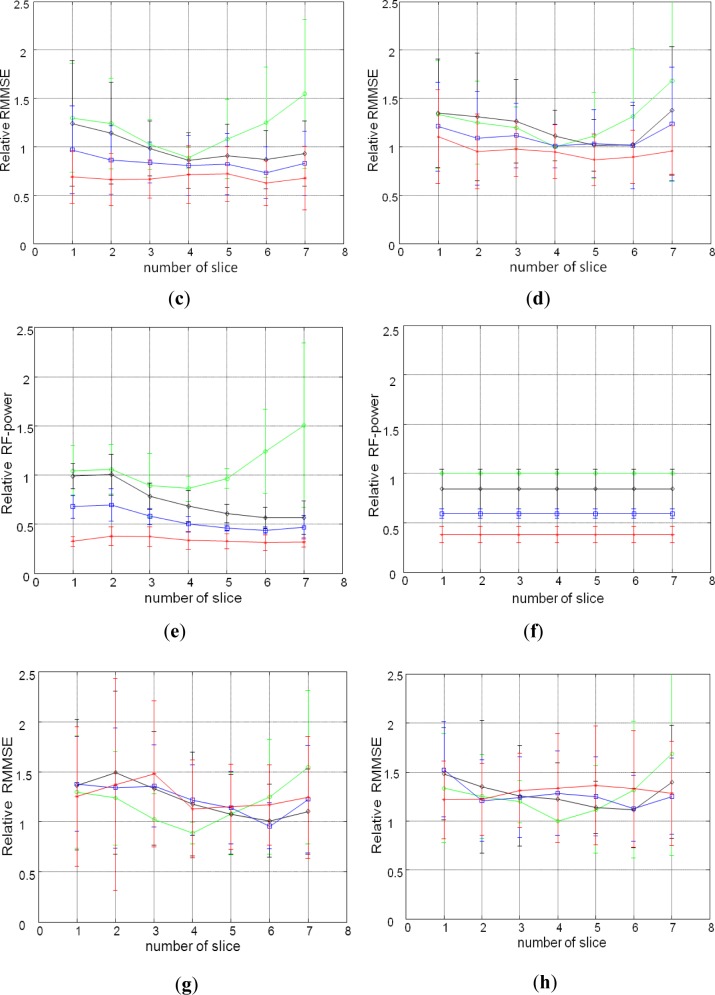

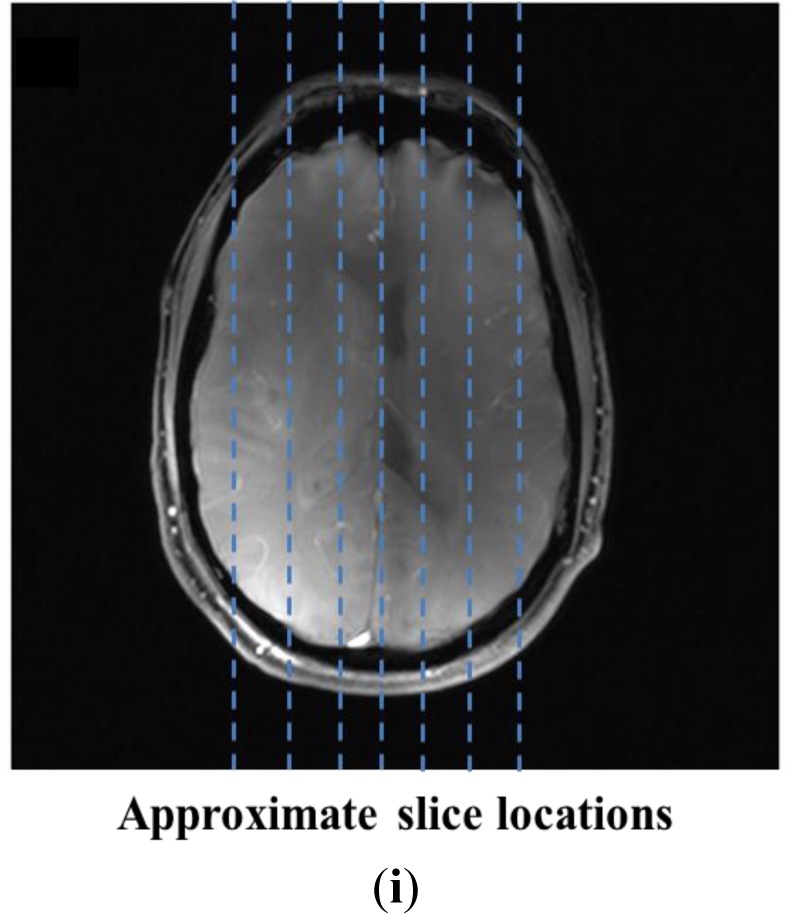
The changes in RF-power and RMMSE (relative to center slice of 1-channel Tx global mode) of B_1_^+^ with slice locations. (**a**–**d**) depicts findings for identical value of RF-power for all Tx channel configurations; (**e**–**h**) shows findings for identical value of RMMSE for all Tx channel configurations and (**i**) localizer image shows approximated sagittal slice locations of human volunteers.

Figure 2 represents changes in the RF-power and RMMSE with slice location for all Tx channel configurations. Results with identical RF-power and identical RMMSE for all channels are represented in Figure 2a–d and 2e–h respectively. All graphs are normalized to the center slice of the 1-channel Tx global mode. For slice-selective RF shimming, each slice was shimmed individually, so the RF-power in Figure 2a changes with slice location, and for global RF shimming, the whole slice stack was shimmed so that Figure 2b depicts the same RF-power for the whole slice stack. For comparison, the RMMSE and RF-power were averaged over the entire slice stack for slice-selective and global RF shimming. The 1-channel Tx global mode has an inferior performance compared with the slice-selective RF shimming method and other Tx channel configurations, with RMMSE in B_1_^+^ being nearly 40% (RMMSE of an ideal B_1_^+^ field would be 0%), and the 1-channel Tx slice-selective mode has an RMMSE of 36%. With constant RF-power and slice-selective RF shimming, the RMMSE for 2-, 4-, and 8-channel Tx when compared with 1-channel Tx (RMMSE of 1-channel Tx scaled to 1%) was 0.82%, 0.7% and 0.56% (reduction in RMMSE was 18%, 30% and 44%) respectively, whereas with a constant RMMSE and slice-selective RF shimming, the RF-power (RF-power of 1-channel Tx scaled to 1%) for 2-, 4-, and 8-channels Tx was 0.69%, 0.51% and 0.31% (reduction in RF-power was 31%, 49% and 69%) respectively, compared with 1-channel Tx. In this scenario, the performance of global RF shimming was inferior compared with the slice-selective method. For identical RF-power, the RMMSE for 2-, 4-, and 8-channels Tx compared to 1-channel Tx was 0.94%, 0.86% and 0.76% (reduction in RMMSE was 6%, 14% and 24%) respectively and the RF-power for identical RMMSE for 2-, 4-, and 8-channels Tx compared with 1-channel Tx excitation was 0.84%, 0.59% and 0.38% (reduction in RF-power was 16%, 41% and 62%) respectively. The slice-selective RF shimming method with 8-channel Tx showed marked improvements over global RF shimming and fewer Tx channels.

**Figure 3.**
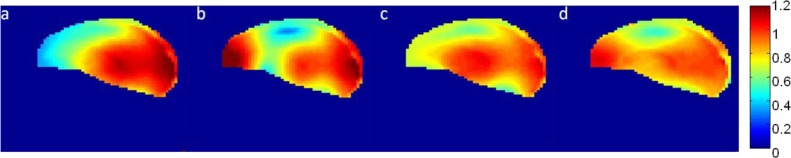
Representative measured sagittal B_1_^+^ maps shimmed with static RF shim, B_1_^+^ maps with four different Tx channel configurations: (**a**) 1-channel Tx; (**b**) 2-channel Tx; (**c**) 4-channel Tx and (**d**) 8-channel Tx.

Examples of the effects of using more Tx channels in a single volunteer can be seen in Figure 3. This is a comparison study for the best (lowest RMMSE) possible homogeneity which can be achieved with 1-, 2-, 4-, and 8-channel Tx using static RF shimming (Figure 3a–d) with identical RF-power for each of the Tx channel configurations. The mean value of all maps was scaled to 1. The magnitude profile resulting from the 2-, 4-, and 8-channel Tx designs depicts improvements in homogeneity of 11%, 27%, and 45% respectively, compared to the 1-channel Tx, which are in the same range for identical RF-power as in (Figure 1). For static RF shimming, the 8-channel Tx design shows significantly reduced RMMSE compared with 1-, 2- or 4-channel Tx shim design.

**Figure 4.**
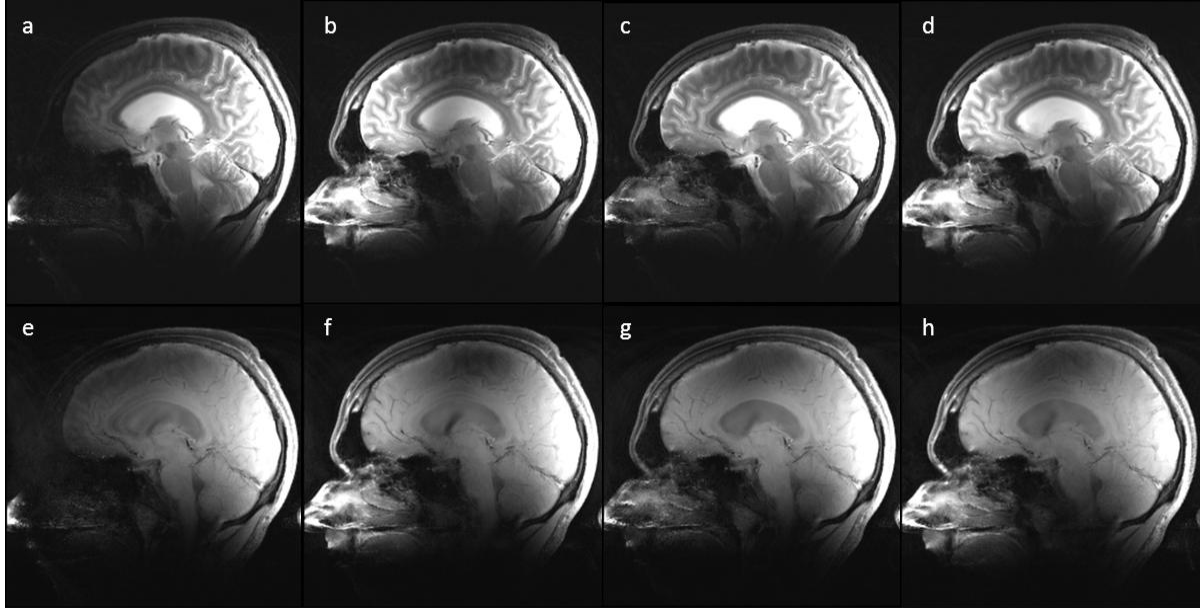
(**a**–**d**) T2w Turbo-Spin-Echo (TSE) and (**e**–**h**) T1w SE images, acquired by performing static RF shimming with 1-, 2-, 4- and 8-channel Tx configurations respectively.

Figure 4 depicts representative slices from the T2w c and the T1w SE image series, acquired with the 1-, 2-, 4-, and 8-channel Tx static shim solutions with identical RF-power. The intensity variations resemble the spatial patterns in the measured B_1_^+^ maps (Figure 3a–d). For the T1w and T2w images, the perceived image homogeneity increases with increasing number of channels for the given brain anatomy. These images were not corrected for received coil sensitivities.

**Figure 5.**
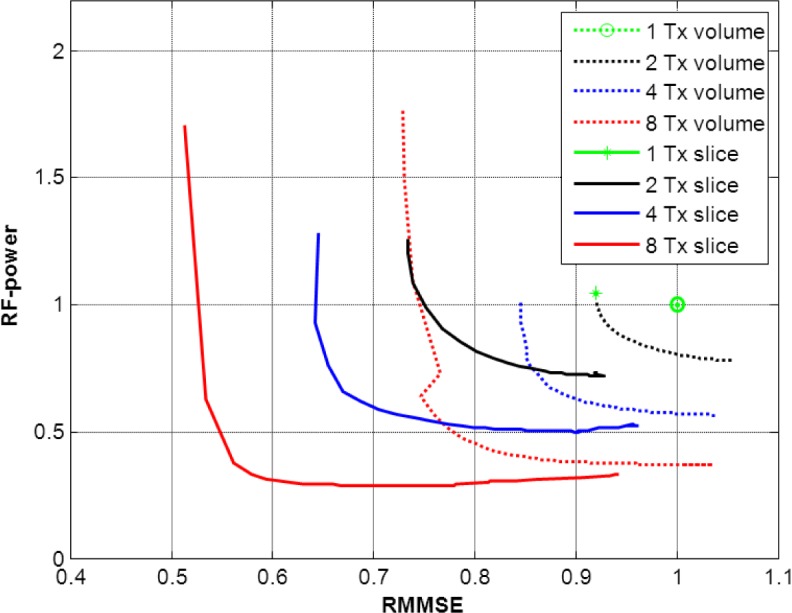
Tradeoffs between relative RF-power and RMMSE for 1-, 2-, 4-, and 8-channel Tx configurations for slice-selective and global RF shimming methods. Each curve is generated by performing the RF shimming calculation for multiple values of the regularization parameter λ. Different colors represent different numbers of Tx channels.

Figure 5 portrays the tradeoff between RF-power and RMMSE when various values of λ (a regularization parameter in Equation (1), explained in the Experimental section) were used for the slice-selective and global RF shimming methods with 1-, 2-, 4- and 8-channel Tx configurations. It shows the inverse relation between RF-power and RMMSE. Imaging experiments were performed for equal RF-power for all Tx channel configurations, where measurable differences in B_1_^+^ were achieved; in order to keep the duration of the examination suitable for the human subjects, the other solutions of λ were not used for imaging. Therefore, the plots presented in Figure 5 are based on simulations.

### Gain in B_1_^+^ Uniformity and Reduction in RF-Power

Using 8 Tx channels and slice-selective RF shimming leads to higher fidelity (by means of reduction ininhomogeneity and RF-power) than the single-channel Tx mode at high field strengths [1]. A standard elliptical birdcage coil together with 1-channel Tx and slice-selective RF shimming yields a RF-power with slight improvement in the RMMSE of B_1_^+^. Therefore, either more sophisticated shimming methods or different Tx coil geometries are required to improve B_1_^+^ homogeneity and/or reduce RF-power over the brain region.

To address this problem, we have used RF shimming with an increasing number of channels (2, 4 and 8), which adds additional degrees of freedom to the B_1_^+^ behavior. With 8 Tx channels, a notable improvement in B_1_^+^ uniformity and/or a reduction in RF-power was observed when compared with a lower number of Tx channels (1-, 2-, or 4-channel Tx). However, this improvement in B_1_^+^ uniformity and/or reduction in RF-power was remarkable even for 2- or 4-channel Tx, when compared with the 1-channel Tx mode. Another approach presented in this work to assess B_1_^+^ behavior was a comparative study of slice-selective RF shimming with the global RF shimming method. In slice-selective RF shimming, each slice was shimmed and scaled up to the desired FA, leading to reduction of RMMSE and RF-power in the slice stack. This reduction in RF-power and RMMSE in the slice-selective RF shimming method was more rapid than for global RF shimming for identical values of λ (Figure 5).

Figure 5 shows that, for small values of λ, the reduction of RF-power is comparatively rapid with only minor increase in RMMSE. With greater values of λ, RF-power reduction becomes negligible and RMMSE increases rapidly. Hence, choosing a specific value of λ in the least square equation at which RF-power reduction ceases to be significant and RMMSE begins to increase could be the efficient solution of λ for RF shimming compared to the standard fixed phase MR system at high field strength which has a major limitation with regards to RF-power. The reduction in RMMSE and RF-power for the slice-selective RF shimming method compared to the global RF shimming method with 2, 4 and 8 Tx channels was 25%, 23% and 20% respectively, for an efficient solution of λ.

Due to the unavailability of a suitable RF-power simulation model for the volunteers imaged for this study, the actual local RF-power values were not modeled. Hence, the imaging performance was restricted to 1/8 (12.5%) of the RF power limits of the same geometry standard single channel transmit and eight channel receive coil (Rapid Biomedical, Rimpar, Germany) to ensure subject safety for any possible constructive RF interference. However, when the system was shimmed with an efficient solution of λ for 8 Tx channels and slice-selective RF shimming, the reduction in RF-power was up to 45% and the improvement inhomogeneity was up to 55%, when compared to a standard single channel Tx and 8 channel receive coil. This RF-power reduction would beneficially increase allowable RF duty cycle, which could be used to increase FA, acquire more slices within a fixed TR or reduce TR. Such effects would be advantageous for sequences requiring higher RF-power, e.g., T1w SE, or for 3D imaging. Slice-selective RF shimming with 8 Tx channels for an efficient solution of λ could also be used in combination with other methods, like Transmit sensitivity encoding [20] or selective excitation [21]. Slice-selective RF shimming would also reduce magnetization transfer effects compare to global RF shimming as these effects depend on the B_1_^+^ field of neighboring slices.

The present work may suggest a systematic study of Tx coil array configurations with multiple elements arranged in all three spatial directions around the head [15,22], and future investigation of additional degrees of freedom which would result in further reduction in inhomogeneity. This study was performed using 8-channels Tx, and the results indicate that RF shimming with more than 8-channels Tx could alleviate B_1_^+^ inhomogeneity even more effectively.

## 3. Experimental Section

All measurements were performed on a 7T MR scanner (Siemens, Erlangen, Germany) with a SC72AB gradient coil (Siemens Erlangen). An oval shaped eight-channel loop coil array (Rapid Biomedical, Rimpar, Germany) was used for transmission and reception. The dimensions of the coil array were: length: 24 cm; width: 28 cm and height: 31.5 cm. The signal was received from the same eight channels in an array reception mode. Parallel transmit hardware was configured to run 8 transmit channels via individual RF amplifiers capable of delivering maximum RF power of 1 kW per channel. This configuration allowed real time control over amplitudes and phases of RF pulse per channel. Worst case RF power limits of 0.15 W (6 min) and 0.30 W (10 s) per channel were derived, based on an assumption that electric fields on all 8 channels superimpose constructively to IEC guidelines [23]. If the power for any channel is exceeded above the limits, the system would immediately shut down the RF power amplifier and stop the sequence, thereby insuring the patient’s safety. The *in vivo* study was approved by the local Institutional Review Board (IRB), and informed consent was obtained from all volunteers before enrollment.

### 3.1. B_1_^+^ Mapping

The B_1_^+^ fields for the eight transmit elements were encoded by applying a slice-selective, Turbo FLASH (TFL) sequence with a saturation pulse applied to a single transmission channel at a time, and received signal from all receive channels independently [24]. The low FA excitation pulses for the TFL readout were transmitted by all channels in the CP phase configuration. This sequence acquired nine scans in a single measurement, the first eight scans with saturation pulses for each of the 8 Tx channels, and the last scan without a saturation pulse. The B_1_^+^ field amplitude was encoded via a magnetization preparation technique: application of a pre-saturation pulse reduces the longitudinal magnetization available for the succeeding imaging sequence according to the local FA of the preparation pulse [25]. Relative phase maps of individual Tx channels were calculated based on low flip angle gradient echo images acquired individually from each Tx channel [1].

Imaging parameters for the 2D multi-slice TFL sequence were: matrix size: 64 × 64; FOV = 220 mm ×220 mm; repetition time (TR) = 5000 ms; echo time (TE) = 1.9 ms, 7 slices, distance factor = 100% and slice thickness = 8 mm, TFL read out FA = 8.

### 3.2. RF Shimming

The CP mode or 1-channel Tx was calculated from the phases of the B_1_^+^ maps such that identical phase of the excitation pattern of all channels in the coil center led to constructive interference. The excitation voltage was identical for each Tx channel. This mimics the behavior of a quadrature birdcage coil, splitting the geometric phase by 2π/c in a c-port cylindrically symmetric coil [26].

Static RF shimming experiments were performed by calculating RF shim solutions for individual Tx channels using the vendor provided magnitude least square optimization algorithm [20]:

$$b=arg_{b}mi\;\{|||Ab|{|}_{w}^{2}+||\lambda b|{|}^{2}\}$$ (1)

The A-matrix incorporates vectors containing the B_1_^+^ profiles of each individual transmit channel; *m* is the desired excitation field an absolute valued vector, for global RF shimming *m* is homogenous; *w* is weighting (shows the optimization was performed over selected region of interest); *b* is the RF shim solution, it contains the complex RF weights and λ denotes a regularization term that may be used to regulate the integrated and peak RF power. In Equation (1), the dimension of *A* is N × N_c_, where N is the number of voxels and N_c_ is the number of individual channel. The dimension of *m* is N × 1 and *b* is an N_c_ × 1 dimensional vector containing the complex coil shim solutions for each Tx channel.

B_1_^+^ maps of 8 individual transmit channels were acquired with CP phase (phases were experimentally adjusted to match a CP solution). Using these B_1_^+^ maps of 8 individual channels, RF shimming adjustments were performed with 2-, 4-, and 8- element combinations. A 2-channel Tx system was mimicked by complex summing of the four adjacent element profiles after CP phase adjustment to form a virtual single coil element. The remaining four adjacent coil elements were summed similarly to form the second coil element. RF shimming was then performed as in a 2-channel Tx situation. Similarly, a 4-channel Tx system was designed with complex summing of two adjacent coil elements and RF shimming experiments were performed on the resulting four-channel transmit system. For the 8-channel Tx system, all Tx channels operated individually for RF shimming.

RF shimming was performed using the magnitude least square algorithm because in most situations the homogeneity of the magnitude profile is of interest, and the phase profile is less relevant provided it varies smoothly to avoid incoherent averaging within one voxel [20]. The vendor provided magnitude least square algorithm, implemented in MATLAB on a desktop PC, calculated all the complex coil element weights for each particular slice and automatically transferred these to the MR system console. Once the complex weights for individual Tx channels and slices were calculated, the sequence used these shim solutions for suitable Tx channels and the slices were acquired.

Simulated B_1_^+^ maps were also obtained for each optimized shim setting to allow comparison between the simulated and measured B_1_^+^ distributions. Based on simulated B_1_^+^ maps, the RF excitation pulses for each measurement were scaled so that the mean FA of experimental results was 90° (nominal), the same as the target FA for all static RF shimming measurements. For slice-selective RF shimming, the mean FA of each slice was adjusted to nominal FA; for the global RF shimming, the mean FA of the whole slice stack was set to nominal the FA. A full examination, consisting of B_1_^+^ mapping, RF shim calculation, and acquisition of images for all channel combinations, could be performed within 50 min.

### 3.3. Comparison of Shim Solutions

The RMMSE method was used to assess the homogeneity of the B_1_^+^ maps. The first term of Equation (1) was used to calculate the deviation of the experimental B_1_^+^ map from the target (m), as shown in Equation (2). In this study, the target pattern m was a homogeneous vector.

$$RMMSE=\sqrt{\||\text{Ab}|-\text{m}{\|}^{2}}$$ (2)

The mean value of B_1_^+^ map was used as the value of target (m) in Equation (2) for calculating RMMSE from the simulation and experimental data, in order to make a fair comparison between both results. The RMMSE value calculated from Equation (2) was divided by the mean value of B_1_^+^ maps. An ideal B_1_^+^ field distribution would have an RMMSE of zero. RF-power in presented work is reported as the sum of squares of RF pulse amplitudes for all channels over all slices in the excited volume. This is a moderate method, which adopts the idea that all transmitted power contributes to RF-power.

As multi slice 2D B_1_^+^ maps were acquired over the brain region, B_1_^+^ shim solutions could be calculated for the individual slices or the full excited volume as one slice stack. To compare different shim techniques, the B_1_^+^ shims were calculated (i) over seven individual slices (slice-selective RF shimming method) and (ii) over the whole slice stack (64 × 64 × 7) that was mapped (global RF shimming method). These two methods were applied to 1-, 2-, 4-, and 8-channel Tx configurations.

Two quantitative studies were performed. The regularization term λ in Equation (1) was used to control the tradeoff between homogeneity and RF-power. In the first study the RF-power of the 2-, 4- and 8-channel Tx was adjusted to be identical to that of the 1-channel Tx system, and the variation in homogeneity for all Tx configurations was analyzed. In the second study, the homogeneity was adjusted to be identical to that of the 1-channel Tx system, and the reduction in RF-power for all Tx configurations was analyzed. These techniques were performed with the slice-selective and global RF shimming methods.

### 3.4. Sequence Protocol

To demonstrate RF shimming performance, T2-weighted 2D Turbo-Spin-Echo (TSE) images and T1-weighted Spin-Echo (SE) images were acquired in sagittal orientation. The imaging parameters for the T2w TSE were FOV: 230 × 230 mm; matrix size: 256 × 256; slice thickness: 5 mm; TR/TE: 5 s/50 ms; echo spacing: 9.93 ms; echoes per train: 9; center echo: 5; and bandwidth: 217 Hz/Px. Imaging parameters for T1w SE were FOV: 230 × 230 mm; matrix size: 256 × 256; slice thickness: 5 mm; TR/TE: 700 ms/9.8 ms; bandwidth: 217 Hz/Px.

## 4. Conclusions

This work demonstrates that increasing the number of independent Tx channels from CP mode to 2-, 4- and 8-channel Tx can improve the homogeneity of the B_1_^+^ field and/or reduce RF-power at high field MR. We have also demonstrated that further homogeneity improvement is possible with 8-channel Tx configurations by using the additional degrees of freedom available in a multi-slice acquisition, and that RF shimming over individual slices is more suited to homogenize and reduce RF-power over the ROI than shimming the entire volume. Shimming over smaller ROIs presents a simpler optimization problem and starts with smaller inhomogeneity, which is an advantage of slice-selective RF shimming. The benefit of using 8 Tx channels and individual slice shimming with an efficient solution of λ is superior RF performance with respect to transmit fidelity compared to 1-channel Tx (CP mode) or global RF shimming. For this study, the maximum number of Tx channels was 8. Hypothetically, superior performance in the brain region could be achieved with a greater number of Tx channels with the use of an appropriate coil array at high field strength.
